# Early psychological intervention in adult patients after hospitalization during COVID-19 pandemia. A single center observational study

**DOI:** 10.3389/fpsyg.2022.1059134

**Published:** 2022-11-16

**Authors:** Elisa Lazzaroni, Davide Tosi, Silvia Pontiggia, Riccardo Ermolli, Luca Borghesi, Vittorio Rigamonti, Enrico Frisone, Stefania Piconi

**Affiliations:** ^1^Department of Mental Health and Addictions, Asst Lecco, Lecco, Italy; ^2^Department of Theoretical and Applied Sciences, Insubria University, Varese, Italy; ^3^Infectious Diseases Unit, Ospedale di Lecco, Asst Lecco, Lecco, Italy; ^4^General Direction, Asst Lecco, Lecco, Italy

**Keywords:** COVID-19 survivors, psychological support, EMDR, hospitalization, prevention

## Abstract

The coronavirus disease 2019 pandemic has represented an individual and collective trauma with an impact on mental health. COVID-19 survivors need to be screened for psychological distress regularly for timely intervention. After March 2020, an outpatients clinic for follow up of discharged COVID-19 patients was set up at Infectious Diseases Department of the Hospital of Lecco, Italy. Blood exams, specialistic visits were performed for each patients and IES-R and BDI scales were dispensed. 523 patients were referred to the clinic; 93 of them resulted positive at IES-R and/or BDI self-report and 58 agreed to have early interviews with psychologist specialist. Patients could receive only a short psychoeducation/psychological support intervention or in addition to the same, even a specific trauma-focused psychotherapeutic intervention with EMDR where clinically indicated. IES-R e BDI were administered pre- and post-intervention. The results show that the average of the post-traumatic stress scores detected at IES-R is above the clinical cut-off for the entire sample. There is an overall change in the decrease in mean scores on the IES and BDI scales before and after psychological intervention. Among the patients for whom psychopharmacological therapy was also necessary, those who had COVID-mourning in family improved the most at IES-R scale post- intervention. With respect to EMDR treatment, there is a significant improvement in depressive symptoms noticed at BDI for male patients who have received neither psychotropic drugs nor CPAP. Being hospitalized for coronavirus has a significant impact on the patient’s mental health and it is a priority to arrange early screening to intercept psychological distress and give it an early response.

## Introduction

The coronavirus disease 2019 (COVID-19) pandemic caused death in 2–5% of COVID patients due to progressive respiratory failure and massive alveolar damage (Xu et al., 2021) and has represented an individual and collective trauma with an impact on mental health (Cucinotta and Vanelli, 2020; Lazzaroni et al., 2021). The entire scientific community has invested both in deeply and promptly analyzing all the available data (Tosi et al., 2020; Tosi and Campi, 2020; Tosi and Campi, 2021; Cappi et al., 2022) beyond developing clinical trials in order to identify new treatments.

Several studies have shown that during the first lockdown measures, in the general population were found high levels of psychological distress such as anxiety, depression, post-traumatic symptoms (Liang et al., 2020, p:1165; Liu et al., 2020; Wang et al., 2020; Xi et al., 2020). Moreover, it was shown that these psychological evidences turn out to be more present in younger patients than in the older. Cai et al. (2020) reported that older COVID-19 survivors have less emotional reactivity to infection, fewer anxiety and stress reaction symptoms than younger survivors.

This psychological impact is even more evident for COVID-19 patients who experienced medium- or high-intensity hospitalization. Patients who required hospitalization with severe physical symptoms and isolation from their family members for a long time, developed and lived concern for their family members’ health, elevated death anxieties, experiences of helplessness and alertness, personal vulnerability, altered sleep patterns, dissociated perception of their body as the object of invasive care actions and negative perceptions of the bodily self, exposure to vicarious trauma (e.g., death of patients admitted to the same ward), experiences of guilt for becoming infected, psychosocial difficulties related to job loss. These conditions are qualified as risk factors for the development of psychological distress or psychopathological symptoms. Special attention should be paid to patients required admission to the intensive care unit (ICU). It is known that these patients are at high risk to develop PTSD, depression, anxiety, sleep abnormalities, and cognitive impairments (McGiffin et al., 2016). A recent meta-analysis found self-reported PTSD symptoms in 24% of ICU patients between one and 6 months after discharge, and 22% at 7 months (Parker et al., 2015; Demiselle et al., 2021). As psychological dysfunction can persist for years after ICU discharge, its management is becoming an important strategy to improve quality of life together with early detection of post-traumatic stress disorder and anxiety and depression (Vlake et al., 2020).

Previous studies conducted on Severe Acute Respiratory Syndrome (SARS) and Middle East Respiratory Syndrome (MERS) outcomes, reported that survivors had shown the prevalence of PTSD, depression, and anxiety beyond 6 months after hospitalization (Ahmed et al., 2020; Shi et al., 2020). In particular, fears, stigma and isolation due to quarantine appeared to be the key determinants of the psychological impact of illness and hospitalization on long-term mental health (Lam et al., 2009). Mazza et al. (2020) screened for psychiatric symptoms 402 adults at 1 month after hospitalization for COVID-19 (265 male, mean age 58): a significant proportion of patients had self-rated in the psychopathological range (28% for PTSD, 31% for depression, 42% for anxiety, 20% for OC symptoms, and 40% for insomnia). Multiple lines of evidence indicate that stress-related disorders including depression, PTSD, and sleep disorders are associated with suicidal ideation, suicide attempts, and death by suicide (Sher, 2019, 2020).

Psychological distress among the COVID-19 survivors in convalescence was high, highlighting the need for all COVID-19 survivors to be screen for psychological distress for timely intervention (Cai et al., 2020).

The aim of this study was to evaluate, through a quantitative analysis, the presence of peri- and posttraumatic stress and depression linked to coronavirus hospitalization experience in followed up patients and to analyze any changes in terms of traumatic stress and levels of depression detected at the end of a brief psychological intervention. It is also intended to test whether there are any correlations between these highlighted changes and demographic variables, intensity of the level of care, and whether psychopharmacological treatment.

## Materials and methods

### Setting

After March 2020, COVID-19 outclinic for discharged patients was set up at Infectious Diseases Department of Alessandro Manzoni Hospital of Lecco, Italy. Blood exams, infectious diseases specialistic visits were performed for each patients and Impact of Event Scale-Revised (IES-R) and Beck Depression Inventory (BDI) questionnaires were dispensed to assess how COVID-19 disease and the hospitalization during the pandemic impacted to their psychological wellness. These type of screening questionnaires were chosen to avoid overloading patients with excessive tests and easy to use by physician. If psychological report was positive, patients were referred to psychologist specialist for a brief cycle of specific interviews after their agreement. At the end of psychological intervention, scales were repurposed to assess any changes. In case of needing of cardiological, neurological, pneumological investigations, the patients were referred to the competent medical specialists.

### Study population

For this study, inclusion criteria were: (i) hospitalization for SARS CoV-2 infection confirmed by nasopharingeal swab RT-PCR and screening visit at outpatient clinic; (ii) positive score at psychological screening self-report; (iii) patients agreement to start brief cycle of psychological support.

Exclusion criteria were: (i) presence of suicidal ideation, severe psychopathological conditions and/or known cognitive deficit.

For each subject, a personal data sheet was compiled, which included demographic information, pharmacotherapy, COVID-19 disease information such as pneumonia severity (mild, moderate or severe) type of oxygen support used (i.e. Continuous Positive Airway Pressure -CPAP or lower intensive oxygen devices); presence of family members died for COVID-19.

### Assessment

After first specialistic visit, patients with positive scores at psychological questionnaires were referred for psychological evaluation. Patients were hospitalized in different departments of A. Manzoni Hospital, according to the severity of COVID-19 disease (i.e., General Medicine, Infectious Diseases, Intensive Care Unit).

A trauma-focused psychological support intervention was proposed to patients with positive scores aimed to facilitating the resumption of the daily life by reducing the impact of this recent traumatic experience. The psychologist who followed the patients is also an experienced trauma-therapist qualified in the use of Eye Movement Desensitization and Reprocessing (EMDR).

#### Study instruments

##### Self-report scales were

*Impact of Event Scale-Revised* (*IES-R*): used to measure stress levels and symptomatology due to the impact of the traumatic event of the pandemic. The IES-R (Weiss and Marmar, 1997) is a 22-item self-report questionnaire consisting of three subscales (eight items relate to intrusions, eight items evaluate avoidance, and six items assess hyperarousal). The scale assesses the subjective distress caused by traumatic events. Participants were asked to rate each item on a scale from 0 (not at all) to 4 (extremely), based on their experience with the traumatic event in the previous 7 days. An IES-R score ≥ 33 represents the best cut-off for a probable diagnosis of PTSD. The IES-R was found to be highly internally consistent (Cronbach’s alpha, α = 0.96; Creamer et al., 2003).

*Beck Depression Inventory* (*BDI*; Beck, 1972): BDI (in the short form of 13 item) is a self-report instrument to assess the severity of depressive symptomatology (Beck et al., 1974). Respondents rated each item based on four response choices according to the severity of the symptoms, ranging from the absence of a symptom to an intense level, during the past week. The score obtained can vary from 0 to 39 (10–19: mild depression; 20–29: moderate depression; >30: severe depression). The BDI maps a wide spectrum of depressive symptomatology (Beck et al., 1961) and features high reliability and validity. Both the original and short forms have reasonable internal consistency for normal and depressed older adults and adequate test–retest reliability in older adult patient and nonpatient populations (Edelstein et al., 2004). Moderate to high correlations show concurrent validity with different depression scales. Albeit no exact value is listed for the diagnosis of a depressive disorder, a comparing statement is possible.

### Intervention

The psychological intervention proposed to patients included a cycle of 4–6 interviews according to progressive modes of intervention. If posttraumatic symptomatology was found to be relevant and intrusive, specific treatment with a few sessions of EMDR was carried out in addition to psychoeducational intervention and psychological support.

The EMDR treatment proposed is according to the *brief EMDR group treatment* protocol created by the re-elaboration of the guidelines for the stabilization-decompression of Critical Incident Stress Management (CISM; Everly Jr. et al., 2001; Quinn, 2009) and the specific EMDR protocols for Acute and Recent Traumatic Events (Shapiro and Laub, 2008). Eye Movement Desensitization and Reprocessing (EMDR) is a therapeutic approach used for the treatment of trauma and traumatic stress-related issues (Shapiro, 2000) based on the Adaptive Information Processing (AIP) model (Shapiro, 2000). According to the AIP, the traumatic event experienced by the subject is stored in memory together with the disturbing emotions, perceptions, cognitions, and physical sensations that characterized that moment. All the information stored in a dysfunctional way remains “frozen” within the neural networks and cannot connect to other networks with useful data (Fernandez and Giovannozzi, 2012); unable to be processed, it continues to cause discomfort in the subject, up to the onset of diseases such as PTSD and other psychological disorders. The aim of EMDR is to restore the natural way of processing the information in the memory to achieve an adaptive resolution through the creation of new, more functional connections. A distinct characteristic of EMDR therapy is the use of alternating bilateral stimulation (e.g., eye movements, tactile stimulation, auditory stimulation, and butterfly hug), which appears to produce a physiological effect promoting accelerated reprocessing of dysfunctionally stored information related to the traumatic event (Jeffries and Davis, 2013; Carletto et al., 2017; Pagani et al., 2017). EMDR is considered as one of the elective psychotherapeutic treatments for PTSD, according to several meta-analyses and clinical guidelines, and its neurobiological effects are also supported by neuroimaging findings (Pagani et al., 2012; Carletto et al., 2018, p: 2). At present, it is recognized as an evidence-based method for the treatment of post-traumatic disorders (Baek et al., 2019; Maddox et al., 2019) approved by the *American Psychological Association* (1998–2002), the *American Psychiatric Association* (2004), the *International Society for Traumatic Stress Studies* (2010), and the *Italian Ministry of Health* in 2003. The WHO in August 2013 recognized EMDR as an effective treatment for trauma and trauma-related disorders.

### Data analysis

Analysis of the collected data was carried out by comparing the averages and resulting delta between the pre and post psychological intervention scores of the IES-R and BDI scales.

IES-R and BDI scale cut offs were 33 and 10, respectively.

One-way analysis of variance (ANOVA, Analysis of Variance) was performed on the whole study population focusing on specific subgroups of patients: psychodrugs assumption started during hospitalization; use of oxigen invasive devices such as CPAP, ICU admission, family mourning for COVID-19. Value of *p* < 0.05 was considered statistically significant. We also conducted a correlation study (Spearman’s correlation) between specific subgroups of patients and variables such as “pre treatmentBDI or IES-R,” “Delta BDI or IES-R,” “pre treatmentBDI vs. BDI post psychodrugs treatment,” etc. However, none of the conducted analysis highlighted moderate or strong correlation levels (i.e., >0.4). For space reason, we do not report the correlation study.

## Results

### Demographic and clinical characteristics of study population

523 hospitalized patients for COVID-19 disease from March 2020 to November 2021 were referred to outpatient clinic for follow up and performed IES-R and BDI questionnaires. 93 (17,7%) of them reported positive scores and 58 (62,3%) agreed to have early interviews with psychologist specialist. Data about age and type of oxigen treatment that meaning COVID-19 severity were reported in Table 1.

**Table 1:** Demographic and oxigen therapy information of patients referred for follow up visit after hospitalization for COVID19.

	**TOT**	**Positive IES-R/BDI scores**	**Positive IES-R/BDI scores agreed psychological treatment**
	***n* = 523**	***n* = 93**	***n* = 58**
**M, n (%)**	344 (65,8)	59 (63,5)	31 (53,4)
**F, n (%)**	179 (34,2)	34 (36,5)	27 (47,5)
**Age, median (IQR)**	65 (55–74)	65 (55–75)	61 (55–69)
**Other oxigen treatment or none, n (%)**	400 (76,5)	63 (68)	28 (48,2)
**CPAP and or ICU, n (%)**	123 (23,5)	30 (32)	30 (51,8)

It is important to observe that the average age (60 years) of our dataset is in line with the median age of Italian population contracting COVID-19 (51 years), requiring the hospitalization (75 years), and requiring ICU (71 years), as ISS (Istituto Superiore di Sanità, 2022) reported.

### IES-R and BDI results

#### Whole population

Table 2 shows the results of pre and post psychological treatment IES-R and BDI scores of the patients of this survey. For both scales analyzed, there is an improvement between the pre and post scores after psychological intervention even not statistical significant.

**Table 2:** Averages values of IES-R and BDI scores of patients agreed psychological treatment and stratified for use or not of psychotropic drug and presence or not of family mourning.

	***N***	**IES pre treatment**	**IES post treatment**	**DELTA IES**	*p* **Value**	**BDI pre treatment**	**BDI post treatment**	**DELTA BDI**	***p* Value**
**All patients**	58	41,03	20,68	20,4	n.s	6,97	5,66	1,31	n.s
**psychotropic drug**	13	45,85	31,31	14,54	n.s	9,23	8,15	1,08	n.s
**without psychotropic drug**	45	39,6	17,58	22,09	n.s	6,31	4,93	1,38	n.s
**psychotropic drug + mourning**	4	53,25	27,5	25,75	**0,03**	12	7,5	4,5	n.s
**without psychotropic drug - mourning**	9	42,56	33	9,56	n.s	8	8,44	-0,44	n.s

The overall clinical changes do not correlate with any of the demographic variables (sex and age) of the patients, from which it appears independent (data not shown).

#### Subgroups population

##### Psychotropic drug administration

It was evaluated the effects of association between pharmacological interventions (anxiolytics as benzodiazepines and/or antidepressants as SSRIs) and psychological interventions on IES-R and BDI differences reported in Table 2. The improvement of patients treated with both interventions appears lower (−28.7% for IES-R and − 17.6% for BDI scores) compared to the improvement of whole study population. In contrast, for patients treated with only psychological interviews IES and BDI scores improvement was higher (+8.3% and + 5.3%, respectively).

##### Psychotropic drug administration and COVID-19 family mourning

Patients treated with psychotropic drugs and that lived COVID-19 family bereavements (Table 2), statistically significant improvement values for delta IES were found (*p* = 0.03) than those who did not have family bereavements. Not statistically difference was reported for BDI scale.

Our data show that family mourning for COVID-19 should be the event associated to the worse pretreatment IES-R score but it resulted to the better recovery as noted in post treatment IES-R score (Delta −25.75).

Regard gender differences, only for female patients treated with psychotropic drugs plus psychological interventions, we observed a significant change between pre and post treatments in IES-R scale compared to not psycodrugs treated-patients (*p* = 0.01, data not shown).

##### Intensity of care (CPAP)

Regarding the intensity of care received, we do not find any significant difference in both psychological scales in patients treated with CPAP device compared to not-treated CPAP patients as showed in Table 3.

**Table 3:** Averages values of IES-R and BDI scores of patients stratified for the use of CPAP device during hospitalization and EMDR treatment during psychological interviews.

	***N***	**IES pre treatment**	**IES post treatment**	**DELTA IES**	*p* **Value**	**BDI pre treatment**	**BDI post treatment**	**DELTA BDI**	*p* **Value**
**CPAP device_total**	29	41,1	21,93	19,17	n.s	6,62	5,62	1	n.s
**Without CPAP device_total**	29	40,1	19,34	21,62	n.s	7,31	5,69	1,62	n.s

##### EMDR treatment (Eye movement desensitization and reprocessing)

In Table 4 it was reported the results of the EMDR intervention in whole population and in different subgroups described above.

**Table 4:** Averages values of IES-R and BDI scores of patients agreed psychological and EMDR treatment with/without use of psychotropic drug.

	***N***	**IES pre treatment**	**IES post treatment**	**DELTA IES**	*p* **Value**	**BDI pre treatment**	**BDI post treatment**	**DELTA BDI**	*p* **Value**
**With EMDR**	29	43,86	22,90	20,97	n.s	7,83	5,41	2,41	n.s
**Without EMDR**	29	38,21	18,38	19,83	n.s	6,10	5,90	0,21	n.s
**Male with EMDR**	17	44,24	22,29	21,95	n.s	8,24	4,88	3,36	**0,033**
**Male without EMDR**	14	33,07	16,50	16,57	n.s	6,29	6,79	-0,50	n.s
**With EMDR + without psychotropic drug**	22	41,23	19,45	21,77	n.s	6,86	4,09	2,77	**0,04**
**Without EMDR + without psychotropic drug**	23	38,13	15,74	22,39	n.s	5,78	5,74	0,04	n.s
**With EMDR + with psychotropic drug**	7	52,14	33,71	18,43	n.s	10,86	9,57	1,29	n.s
**CPAP device + EMDR**	19	44,42	25,53	18,89	n.s	7,47	6,42	1,05	n.s
**CPAP device - EMDR**	10	34,80	13,30	21,50	n.s	5,00	3,60	1,40	n.s
**Without CPAP device + EMDR**	10	42,80	17,90	24,90	n.s	8,50	3,50	5,00	**0,011**
**Without CPAP device - EMDR**	19	40,00	20,11	19,89	n.s	6,68	6,84	-0,16	n.s

In whole population we found better improvement in both psychological questionnaires in patients treated with EMDR intervention compared to not treated patients, despite not statistically significant (Delta 20.97 and 2.41 for IES-R and BDI scale, respectively).

Among patients not treated with psychotropic drugs, we found a statistical difference in BDI scale values between patients treated with EMDR compared to not EMDR treated patients (*p* = 0.04; Table 4). Moreover, it was noted the better improvement in BDI scale compared to the whole population (delta BDI +111.5%). Among patients treated both with psychotropic drugs and EMDR (7 patients out of 29), we found a non-significant Delta of IES-R (Delta 18.43) and BDI (Delta 1.29). These variations are lower than the ones observed for patients treated with EMDR but without psychotropic drugs.

In not EMDR treatment patients, pretreatment scores for BDI scale are slightly lower than the mean of whole study population but BDI delta is almost nil.

In CPAP treated patients we found better improvement in both psychological questionnaires in patients treated with EMDR intervention compared to not EMDR treated patients, despite not statistically significant (Delta 24.9 and 1.05 for IES-R and BDI scale, respectively). Significant positive results, especially for BDI scores, were found in mild COVID-19 disease patients (not CPAP treated patients) after EMDR intervention (*p* = 0.011) compared to not EMDR treated patients (Table 4). The same trend was found for IES-R scores, despite not statistically significant.

Regard gender differences, male patients treated with EMDR intervention showed a statistically significant improvement in BDI scores (Table 4), *p* = 0.033.

## Discussion

In the whole study population analyzed, it is possible to observe that the mean scores of post-traumatic stress detected at the IES-R are above the clinical cut-off for the entire sample considered, highlighting how SARS-CoV-2 infection might have a significant impact on a patient’s mental health (Shi et al., 2020; Xiong et al., 2020; Lazzaroni et al., 2021). There is an overall quantitatively detected change in decreasing mean scores on the IES and BDI scales pre and post psychological intervention, suggesting that early psychological interventions may contribute positively to the reduction of psychological distress caused by traumatic events, as coronavirus disease 2019, as reported extensively in different systematic reviews and meta-analyses (Roberts et al., 2019). The result is particularly interesting if we consider that the psychological intervention was very short (4–6 interviews). It is also interesting to note that in this survey patient adherence to psychological intervention was very high (64%).

No statistically significant correlations emerged between the demographic variables and the changes found in the sample considered.

In our study population that included only patients older than 40 years (mean age: 61 years), it is not possible to demonstrate greater improvements in younger patients due to this type of patients were not generally hospitalized for COVID-19. Despite some recent studies reported that younger people seemed to have better coping skills (Buzzi et al., 2020; Kar et al., 2021), subjects interviewed in this study had the same abilities to overcome adverse living conditions experienced during the COVID-19 pandemic.

Regarding the assumption of psychotropic drugs, for female patients there is a significant change between psychotropic drugs treated patients compared to who did not receive such therapy. IES-R values were above the cut-off of that scale for both subgroups of patients, but remain above the threshold only for patients who assume psychotropic drugs, in contrast to those who did not use psychotropic drugs that reported a drastic drop in IES post-treatment. Female patients in fact have suffered more the traumatic impact of COVID-19-related events compared to males (Xiong et al., 2020.) and, as consequence, they more often received a psychopharmacological therapy. For those patients, the only brief psychological intervention was deemed insufficient for patient care (Lill, 2015; Zhou et al., 2017). Among the patients that required psychopharmacological therapy, those who presented family Covid-19 mourning improve the most in IES scale score post- psychological intervention. It is possible hypothesize that COVID-19 family bereavements may be considered a factor of greater impact in terms of post-traumatic stress and depressive experiences on patients then hospitalized for COVID-19 (such that psychopharmacological therapy is also needed), but psychological intervention contributes more to psychological recovery facilitating the processing of prior bereavement, having untied the clinical “knot” of unprocessed bereavement.

Regarding severe COVID-19 disease in our sample, despite of what reported in literature (McGiffin et al., 2016), we do not find any significant difference in both psychological scales in patients treated with CPAP device compared to not-treated CPAP patients. The experience of severe Sar-Cov-2 infection such that one needs hospitalization (both ordinary admissions and ICU admissions) with the related death risk perceived, it is an experience that generates high traumatic distress regardless the intensity care received. However, it is shown (Table 4) specifically among patients treated with EMDR and not required CPAP, they improve more than the remaining clinical population, specifically with regard to depressive symptomatology detected on the BDI scale. In particular, for patients treated with CPAP device there is less improvement in depressive symptomatology compared to who was not treated with the device. This observation allows us to consider that focal treatment with EMDR may result in a more positive clinical change for patients who had not experienced complex forms of Intensive Care Unit, promoting a more rapid recovery for this category of patients.

We observe a positive improvement in depressive symptomatology detected by the BDI scale for the following categories of EMDR treated patients: patients treated with psychopharmacological therapy, patients not treated with CPAP device, and male patients. Conversely, EMDR not-treated patients had post-treatment values for the BDI scale equal to or greater than pre-treatment values. We also found better improvement in IES-R scales in patients treated with EMDR intervention compared to not treated patients, despite not statistically significant.

It is thus clear that EMDR treatment played, for these subgroups, a key role in improvement with regard to depressive symptomatology. EMDR is a well-established treatment for post-traumatic stress disorder (Cuijpers et al., 2020). Although recent research suggested that it may be effective in treating depressive disorders as well (Hofmann et al., 2014). These findings confirm earlier suggestions that EMDR therapy may provide additional benefit in the treatment of depression (Hase et al., 2018).

### Study limitations

The main limitation of the study concerns the very small sample size and the high average age of the sample. It is also possible to highlight among the limitations the absence of control group with patients who did not receive any psychological intervention.

Another limitation is the absence of a self-report scale to detect psychopathological diagnoses of any instrument for general psychopathology.

## Conclusion

The coronavirus disease 2019 (COVID-19) pandemic has represented an individual and collective trauma with an impact on mental health. There is an overall quantitatively detected change in the decrease in mean scores on the IES and BDI scales pre- and post-psychological intervention, suggesting how early psychological interventions can contribute positively to the reduction of psychological distress caused by traumatic events, as coronavirus disease 2019.

Intensity of care is not a determinant factor for post-traumatic stress and depressive symptoms in hospitalized patients. The improvement of patients treated with both pharmacological and psychological interventions appears lower compared to the improvement of whole study population. Among the patients needing psychopharmacological therapy, those who had COVID-19 family mourning improved the most at IES-R scale post- intervention.

With respect to EMDR treatment, there is a significant improvement in depressive symptoms noticed at BDI for male patients who have received neither psychotropic drugs nor CPAP. It would be interesting in the future to investigate whether with a greater number of interviews it would have been possible to obtain more significant results on the IES scale for patients treated with EMDR.

It appears to be a priority to carry out early screening of possible post-traumatic and depressive symptoms in order to intercept psychological needs to treat by setting up early psychological support interventions and promote the physiological resumption of life routines.

## Data availability statement

The raw data supporting the conclusions of this article will be made available by the authors, without undue reservation.

## Ethics statement

Ethical review and approval were not required for the study on human participants in accordance with the local legislation and institutional requirements. The patients/participants provided their written informed consent to participate in this study.

## Author contributions

RV and SPi: conceptualization, study design, methodology, writing—review and editing. EL: methodology, data collection, formal analysis, and writing—original draft. SPo and LB: data collection and organization. DT and RE: statistical analysis. EL, SPo, and SPi: development of literature review/background context, manuscript review and editing. VR, SPi, and EF: resources and supervision. All authors contributed to the article and approved the submitted version.

## Conflict of interest

The authors declare that the research was conducted in the absence of any commercial or financial relationships that could be construed as a potential conflict of interest.

## Publisher’s note

All claims expressed in this article are solely those of the authors and do not necessarily represent those of their affiliated organizations, or those of the publisher, the editors and the reviewers. Any product that may be evaluated in this article, or claim that may be made by its manufacturer, is not guaranteed or endorsed by the publisher.
